# Comparative studies of C_3_ and C_4_ *Atriplex* hybrids in the genomics era: physiological assessments

**DOI:** 10.1093/jxb/eru106

**Published:** 2014-03-27

**Authors:** Jason C. Oakley, Stefanie Sultmanis, Corey R. Stinson, Tammy L. Sage, Rowan F. Sage

**Affiliations:** ^1^Department of Ecology and Evolutionary Biology, The University of Toronto, 25 Willcocks Street, Toronto, ON M5S3B2Canada

**Keywords:** C4 engineering, C4 photosynthesis, CO2 concentrating mechanism, photosynthetic hybrids, Rubisco.

## Abstract

We crossed the C_3_ species *Atriplex prostrata* with the C_4_ species *Atriplex rosea* to produce F_1_ and F_2_ hybrids. All hybrids exhibited C_3_-like δ^13^C values, and had reduced rates of net CO_2_ assimilation compared with *A. prostrata*. The activities of the major C_4_ cycle enzymes PEP carboxylase, NAD-malic enzyme, and pyruvate-P_i_ dikinase in the hybrids were at most 36% of the C_4_ values. These results demonstrate the C_4_ metabolic cycle was disrupted in the hybrids. Photosynthetic CO_2_ compensation points (Г) of the hybrids were generally midway between the C_3_ and C_4_ values, and in most hybrids were accompanied by low, C_3_-like activities in one or more of the major C_4_ cycle enzymes. This supports the possibility that most hybrids use a photorespiratory glycine shuttle to concentrate CO_2_ into the bundle sheath cells. One hybrid exhibited a C_4_-like Г of 4 µmol mol^–1^, indicating engagement of a C_4_ metabolic cycle. Consistently, this hybrid had elevated activities of all measured C_4_ cycle enzymes relative to the C_3_ parent; however, C_3_-like carbon isotope ratios indicate the low Г is mainly due to a photorespiratory glycine shuttle. The anatomy of the hybrids resembled that of C_3_-C_4_ intermediate species using a glycine shuttle to concentrate CO_2_ in the bundle sheath, and is further evidence that this physiology is the predominant, default condition of the F_2_ hybrids. Progeny of these hybrids should further segregate C_3_ and C_4_ traits and in doing so assist in the discovery of C_4_ genes using high-throughput methods of the genomics era.

## Summary

Leaf anatomy and physiology are characterized in newly generated hybrids between C_3_ and C_4_ species of *Atriplex*, thus re-establishing the classic system exploited by Björkman and colleagues 45 years ago.

## Introduction

C_4_ photosynthesis is a carbon-concentrating mechanism that evolved from C_3_ progenitors at least 65 times (Sage *et al.*, 2012). During C_4_ evolution, a coordinated series of anatomical and biochemical adjustments established the compartmentation and enzyme activities required to efficiently concentrate CO_2_ around Rubisco (Monson and Rawsthorne, 2000). In the process, dozens to hundreds of genes have been altered (Bräutigam *et al.*, 2011a, b; Gowik *et al.*, 2011). A number of the modifications to key biochemical enzymes such as PEP carboxylase have been identified, although most remain unknown, particularly the genes controlling the anatomical modifications (Kajala *et al.*, 2011; Ludwig, 2013). Identification of these elements is essential in the effort to improve C_4_ photosynthesis and potentially engineer the C_4_ pathway into C_3_ crops, as is now being attempted with rice (von Caemmerer *et al.*, 2012; http://c4rice.irri.org/).

**Fig. 1.**
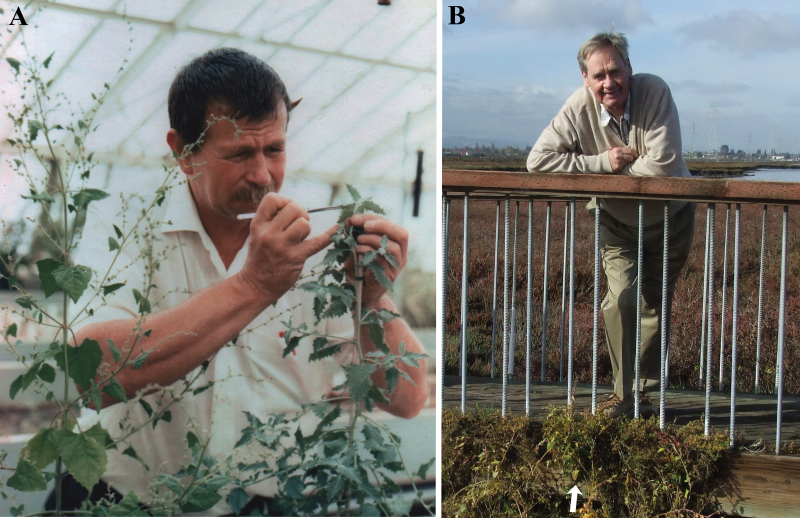
(A) Malcolm Nobs pollinating *Atriplex rosea*, with the pollen donor, *Atriplex prostrata*, to his right. Photo supplied by Olle Björkman, with kind permission. (B) Olle Björkman standing behind a clump of *Atriplex prostrata* (arrow) at the collection site, December 15, 2010 (Photo by R.F. Sage). (This figure is available in colour at *JXB* online.)

Gene discovery is most efficient when researchers can apply forward and reverse genetic approaches using genetic model organisms (Meinke *et al.*, 1998). Unfortunately, in the case of the C_4_ pathway, ideal model organisms have not been developed, although *Setaria viridis* is a potential candidate (Li and Brutnell, 2011; Covshoff *et al.*, 2014). The lack of tractable genetic models for C_4_ photosynthesis requires that alternative means of gene discovery be considered. One option is to generate hybrids between closely related C_3_ and C_4_ species, and then use a genetic mapping strategy to associate genes with segregating traits. A number of congeneric pairs of C_3_ and C_4_ species have been hybridized since the discovery of the C_4_ pathway. The first C_3_ × C_4_ hybrids were produced by Malcolm Nobs and Olle Björkman (Fig. 1) between *Atriplex rosea* (C_4_) and *Atriplex prostrata* (C_3_, formerly termed *A. patula* ssp. *hastata* and *A. triangularis*; Kadereit *et al.*, 2010), and *A. rosea* and *A. glabriuscula* (C_3_) (Björkman *et al.*, 1969; Osmond *et al.*, 1980). Subsequent efforts created hybrids between C_3_ and C_4_-like *Flaveria* species (Apel *et al.*, 1988), and C_3_-C_4_ intermediate and C_4_ *Flaveria* species (Brown *et al.*, 1986; Brown and Bouton, 1993). Hybrids have also been generated between C_3_ and C_3_-C_4_ intermediate *Panicum* species (Bouton *et al.*, 1986). In many of the *Flaveria* crosses, the F_1_ hybrids were sterile (Brown and Bouton, 1993). In cases where F_2_ hybrids were generated and segregation of traits observed, problems associated with chromosome abnormalities and pairing were evident, such that mapping populations could not be formed (Osmond *et al.*, 1980; Covshoff *et al.*, 2014). All hybrid studies were abandoned, and the hybrids eventually perished.

With the advent of high-throughput sequencing and bioinformatics, the ability to evaluate genetic differences between hybrid offspring has dramatically improved, such that the requirement for a mapping population can be relaxed. Of particular promise is sequencing of transcriptomes (RNA-Seq), which can quantify gene expression over a large dynamic range and does not require prior knowledge of the genome sequence (Bräutigam and Gowik, 2010). Comparative transcriptomics has already been used to identify genes that are differentially expressed in leaves of closely related C_3_ and C_4_ plants (Bräutigam *et al.*, 2011a, b; Gowik *et al.*, 2011). By using a comparative transcriptomics approach with segregating F_2_ hybrids, the C_4_ genes controlling the segregating traits may be identified.

C_3_ × C_4_ hybrids can also provide novel insights for understanding C_4_ structure, function, and evolution. With advances in photosynthetic methodology, the development of theoretical models of C_3_ and C_4_ photosynthesis, and an improved appreciation of how structural adaptations enhance C_4_ function, we are now in a much better position to interpret patterns observed in C_3_ × C_4_ hybrid lines then was the case a generation ago (Dengler and Nelson, 1999; von Caemmerer, 2000; Sage *et al.*, 2013). Predictions from theoretical models of C_4_ photosynthesis developed since the hybrid era can also provide valuable insights that will aid the interpretation of C_3_ × C_4_ hybrid studies (von Caemmerer, 2000; Ubierna *et al.*, 2013). In addition, models describing the function of C_3_-C_4_ intermediate species (Rawsthorne *et al.*, 1988; von Caemmerer, 1989 and 1992) appeared near the end of the hybrid studies (Brown and Bouton, 1993). With the modern understanding of C_3_-C_4_ intermediacy, it is now possible to address the degree to which C_3_ × C_4_ hybrids express the physiology of C_3_, C_4_, or C_3_-C_4_ intermediate species (Sage *et al.*, 2012). In C_3_-C_4_ intermediates, the major physiological trait is a CO_2_-concentrating mechanism (CCM) that shuttles photorespiratory glycine from mesophyll (M) to bundle sheath (BS) tissues where the photorespiratory enzyme glycine decarboxylase is localized (Monson and Rawsthorne, 2000). This CCM is now termed C_2_ photosynthesis (Sage *et al.*, 2012).

In reviewing the literature on C_3_ × C_4_ hybrids, the most attractive system seems to be the cross between *A. rosea* and *A. prostrata* (Björkman *et al.*, 1969). An appealing aspect of this system is that the axile inflorescences of *A. rosea* are entirely composed of female flowers. This facilitates cross-pollination with *A. rosea* as the maternal parent because the bisexual inflorescences at the branch tips can be easily removed (Osmond *et al.*, 1980). The F_1_ offspring of the *A. rosea × A. prostrata* cross are fertile, although with reduced pollen fertility and seed set. The F_2_ offspring exhibit a gradation in many C_4_ traits, with independent assortment (Boynton *et al.*, 1970). For example, no correlation is apparent between leaf anatomy and expression of C_4_ enzymes (Boynton *et al.*, 1970). These findings were the first to demonstrate that multiple genes are involved in the expression of C_4_ photosynthesis, and show that the loss of any one C_4_ trait leads to breakdown of the C_4_ CCM (Björkman, 1976; Osmond *et al.*, 1980). However, chromosomal abnormalities were observed, with only four out of nine chromosomes regularly pairing at meiosis (Nobs, 1976). This precluded traditional genetic analysis, as forming a linkage map was impossible. The use of high-throughput genomics can potentially overcome this constraint (Bräutigam and Gowik, 2010).

To exploit the potential of C_3_ × C_4_ hybrids in the genomics era, it is necessary to produce new hybrid lines to replace those lost decades ago. We therefore regenerated hybrids between *A. rosea* and *A. prostrata* through to the F_2_ generation. Here, we describe the physiology and leaf anatomy of these hybrids using gas exchange and biochemical assays, and interpret the results in light of current theory for the function of C_3_-C_4_ intermediate and C_4_ systems.

## Materials and methods

### Generation of F_1_ and F_2_ hybrids

With the assistance of Olle Björkman (Fig. 1B), seeds of *A. prostrata* were collected from a salt marsh along San Francisco Bay in Baylands Park, Palo Alto, California USA (37°27’38.65”N × 122°06’19.63”W). This is the same collection site for this species in the first hybrid trials (Björkman *et al.*, 1969). Seeds of *A. rosea* were collected in a corral along Ball’s Canyon road, 30 km northwest of Reno, Nevada, USA by Chris Root (39°39’20.68”N × 120°03’19.89”W). All plants used for crosses were grown from these collections in a rooftop greenhouse located at the University of Toronto. Plants were grown in a mixture of sand, Pro-Mix (Premier Tech Ltd., Rivière-du-Loup, Québec, Canada), and sterilized topsoil (2:2:1 by volume) in either 7.6 l or 3.8 l pots. Plants were watered as necessary to avoid drought and fertilized weekly with a mixture containing 1.8g l^–1^ of 24-8-16 Miracle-Gro All Purpose fertilizer, 1.2g l^–1^ 30-10-10 Miracle-Gro Evergreen Tree and Shrub fertilizer (Scotts Miracle-Gro Co., Marysville, Ohio, USA), 4.0mM Ca(NO_3_)_2_, and 1.0mM MgSO_4_. The daytime temperature during growth was 26– 32 ºC depending on outdoor temperature and solar insolation, and night temperature was approximately 23 ºC.

In *A. rosea*, bisexual inflorescences are produced at the branch tips, whereas only female inflorescences are produced in the leaf axils of mature stems (Osmond *et al.*, 1980). *A. prostrata* has only bisexual inflorescences. By removing the bisexual inflorescences from *A. rosea,* we were able to protect the axile flowers from self-pollination and ensure they would receive only pollen produced by *A. prostrata.* Flowers of *A. rosea* were pollinated using an extra-fine paintbrush from August to October, 2011. F_1_ hybrid seed was mature when plants senesced in mid-to-late November, 2011. F_1_ hybrids were grown in identical environments as the parents, using high-pressure sodium lamps to maintain photoperiod at 14h. These plants flowered beginning in mid-August and were allowed to self-pollinate, with seeds maturing by late October.

The F_2_ hybrids, along with F_1_, *A. rosea* and *A. prostrata* plants were grown in a plant growth chamber (Conviron PGC-20, Conviron Ltd., Winnipeg, Manitoba, Canada) at 27 ºC day/22 ºC night using the same soil, watering, and fertilizer regime as described above. Photoperiod was 18h with a light intensity near 700 µmol m^–2^ s^–1^ during the central 8-h portion of the photoperiod, and 200 µmol m^–2^ s^–1^ for 4h on each side of the high light period. One hour of incandescent light provided 20 µmol m^–2^ s^–1^ during the first and last hour of the photoperiod. We selected this photoperiod after preliminary trials showed plants flowered in a 14h photoperiod.

### Gas exchange, leaf nitrogen, and enzyme assays

Gas exchange measurements were conducted on 6–10-week-old plants, using a recently expanded leaf for all measurements. Leaf disks for enzyme and nitrogen assays were sampled from the leaves used for gas exchange. Carbon isotope ratios of leaf disks from adjacent leaves were determined by the University of Washington Isotope Facility (http://depts.washington.edu/isolab/). Whole-leaf gas exchange parameters were measured using a LI-6400 portable photosynthesis system (Li-Cor, Inc., Lincoln, Nebraska, USA) at a leaf temperature of 30 ºC (Vogan *et al.*, 2007). For determination of the response of net CO_2_ assimilation rate (*A*) to intercellular CO_2_ content (*C*_*i*_), a saturating light intensity of 1500 µmol m^–2^ s^–1^ was used for *A. prostrata* and 1800 µmol m^–2^ s^–1^ for *A. rosea*. In the measurement of the *A*/*C*_*i*_ response, leaves were first equilibrated to saturating light (1500 µmol m^–2^ s^–1^ for *A. prostrata* and 1800 µmol m^–2^ s^–1^ for *A. rosea*) and then measurements were recorded. Subsequently, ambient CO_2_ concentration was raised to almost 1000 µmol mol^–1^ to determine the maximum assimilation rate and then reduced in steps to 35 µmol mol^–1^ for *A. prostrata,* 10 µmol mol^–1^ for *A. rosea* and 20 µmol mol^–1^ for the F_1_ and F_2_ hybrids. The CO_2_ compensation point was calculated using the x-intercept of a linear regression through the lowest 4–6 CO_2_ concentrations that fell on a linear response of *A* versus *C*_*i*_. This regression was also used to calculate the initial slope of the *A*/*C*_*i*_ curve, which is an estimate of carboxylation efficiency (CE). Leaf nitrogen was assayed using a Costech ESC 4010 C:N analyzer by the University of Nebraska Ecosystem Analysis lab, Lincoln, Nebraska (biosci.unl.edu/facilities).

Enzyme assays were conducted at 30 ºC for Rubisco and three C_4_ cycle enzymes: phosphoenolpyruvate carboxylase (PEPCase), NAD malic enzyme (NAD-ME), and pyruvate phosphate dikinase (PPDK) (Sage *et al.*, 2011). Leaf samples were extracted into 50mM HEPES buffer (pH 7.8) containing 10mM MgCl_2_, 2mM MnCl_2_, 1mM EDTA, 2% PVPP (w/v), 1% PVP, 1% BSA, 10mM DTT, 0.5% (v/v) Triton X-100, 10mM 6-aminocaproic acid, and 2mM benzamide. Enzyme activities were assayed with a diode array spectrophotometer by measuring at 340nm the reduction of NAD^+^ (for NAD-ME), or the oxidation of NADH in a coupled enzyme assay (Rubisco, PEPCase, PPDK). NAD-malic enzyme and PEP carboxylase were assayed according to Sage *et al.*, (2011). Rubisco was assayed according to Ashton *et al.* (1990), with the extract being incubated in the reaction mixture for 10min before the assay to ensure full activation of the enzyme. The PPDK assay was modified from Ashton *et al.* (1990), with 10mM KHCO_3_ replacing NaHCO_3_ and the concentration of PEPCase being increased to 3 units ml^–1^. All chemicals for enzymes assays with the exception of PEPCase were obtained from Sigma-Aldrich, St. Louis, USA. PEPCase was obtained from Bio-Research Products, North Liberty, Iowa, USA.

### Leaf anatomy

For light and transmission microscopy, 2mm^2^ samples were cut from the middle region of recently expanded leaves and prepared for microscopy as described by Sage and Williams (1995). Briefly, sections were fixed in 2% glutaraldehyde and 0.5M sodium cacodylate buffer solution (pH 6.9) and post-fixed with a 2% osmium tetroxide solution. Samples were then dehydrated in ethanol increments and embedded in Spurr’s resin. The microscopy samples were obtained from leaves adjacent to those used for gas exchange analyses, and were harvested in the middle of the four-week period when gas exchange data were acquired.

## Results

### Generation and growth of the F_1_ and F_2_ hybrids

Approximately 80% of *A. rosea* flowers that were hand-pollinated with *A. prostrata* pollen yielded seed. By contrast, Nobs *et al.*, (1970) reported seed set near 10%. Seedlings of F_1_ plants were easy to identify as they lacked the red colour present on the bottom of *A. rosea* leaves. The F_1_ hybrids produced 50–100 F_2_ seeds each, similar to the results of Nobs *et al.*, (1970). The germination rate for F_1_ seeds was over 80%. The growth habit and leaf shape of the F_1_ hybrids was intermediate between that of the parents and uniform compared with each other, whereas the F_2_ hybrids were also intermediate in growth habit, but exhibited variable leaf shape (Supplementary Fig. S1). Notably, all F_1_ and F_2_ hybrids retained female-only inflorescences in the leaf axils, as seen in the maternal parent *A. rosea.*

## Gas exchange results

**Fig. 2.**
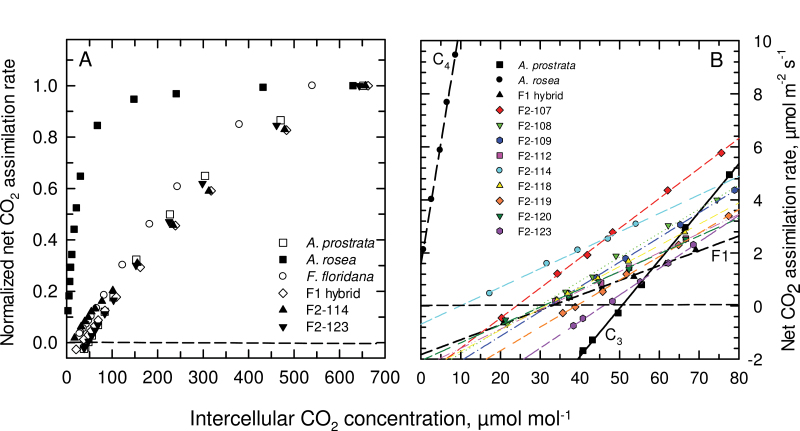
The response of net CO_2_ assimilation rate, *A*, to intercellular CO_2_ (*C*_*i*_) at 30 °C and 1500 µmol photons m^–2^ s^–1^ for the two *Atriplex* parents, an F_1_ hybrid and F_2_ hybrids. (A) Normalized net CO_2_ assimilation rate for the *Atriplex* parents, the C_3_-C_4_ intermediate *Flaveria floridana*, an F_1_ hybrid, and the F_2_ hybrids 114 and 123. (B) The low CO_2_ portion of the *A* versus *C*_*i*_ response illustrating CO_2_ compensation points and initial slopes for all hybrids in the study. Results shown are representative responses of 3–6 *A* versus *C*_*i*_ measurements for the hybrids and *A. prostrata*, and two measurements of *A. rosea*. See Supplementary Fig. S2 for the non-normalized *A*/*C*_*i*_ responses of each hybrid.

**Fig. 3.**
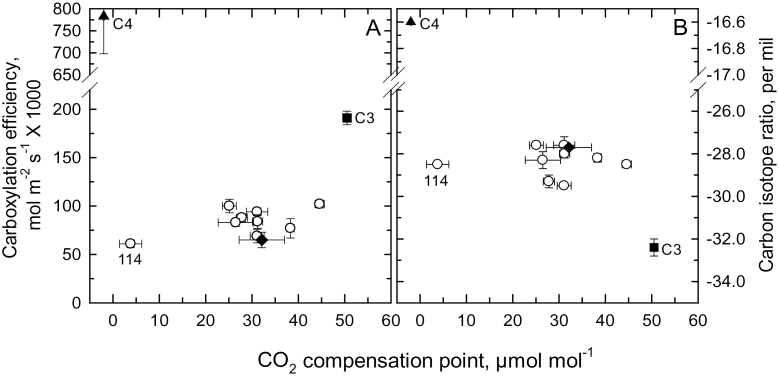
The relationship between the CO_2_ compensation point of the net CO_2_ assimilation rate and (A) the carboxylation efficiency of photosynthesis and (B) the carbon isotope ratio of leaves in *Atriplex prostrata* (C_3_, ■), *Atriplex rosea* (C_4_, ▲), an F_1_ hybrid (♦), and F_2_ hybrids (●). “114” indicates the datapoint for the F_2_-114 hybrid. Some error bars are obscured by the symbols.

The F_1_ hybrids exhibited a CO_2_ compensation point (Γ) near 30 µmol mol^–1^, in contrast to nearly 0 µmol mol^–1^ in *A. rosea* and 50 µmol mol^–1^ in *A. prostrata*, at 30 °C (Fig. 2). Representative *A*/*C*_*i*_ responses for the parents and all hybrids are presented in Supplementary Fig. S2. In Fig. 2A, we show normalized *A*/*C*_*i*_ responses of the C_3_ and C_4_ parents, three hybrids, and for comparison, the C_3_-C_4_ intermediate species *Flaveria floridana*. The normalized curves demonstrate the F_1_ and F_2_ hybrids had a similar qualitative response as *A. prostrata* and *F. floridana*, with the major exception being that the hybrids had a lower carboxylation efficiency (CE) and CO_2_ compensation point (Γ) than *A. prostrata* (Fig. 2B; Fig 3A). The Γ values of the F_2_ hybrids ranged from a C_4_-like value of 4 µmol mol^–1^ in F_2_-114 to 45 µmol mol^–1^ in F_2_-123; Γ in most F_2_ hybrids clustered between 25–35 µmol mol^–1^ (Table 1; Fig. 3). At current air levels of CO_2_ (about 400 µmol mol^–1^ in Toronto), *A*_400_ values in the F_2_ hybrids ranged between 48% and 67% (average 57%) of the *A. prostrata* value (Table 1). At CO_2_ saturation, the difference between the mean *A* value (*A*_*max*_) of the F_2_ hybrid lines and *A. prostrata* was less: *A*_*max*_ in the hybrids ranged between 68% and 89% (mean 77%) of the C_3_ values (Table 1). The difference in the *A*_400_ values between the hybrids and *A. prostrata* was largely due to reduced carboxylation efficiency in the hybrids. The CE values ranged from 32–53% (average 44%) of the C_3_ value in the F_1_ and F_2_ hybrids (Table 1), and exhibited no relationship with variation in Γ (Fig 3A). The δ^13^C of the F_1_ and F_2_ hybrids ranged from –29.3 to –27.6‰, and were consistently more positive than the C_3_ mean of –32.2‰ (Fig. 3B). These values were shifted more negative by approximately 2‰ units owing to an enriched fossil fuel signature in downtown Toronto, where the growth facilities are located. No relationship was apparent between δ^13^C and either Γ, *A*_400_, or *A*_*max*_, and the CE value (not shown).

**Table 1.** Summary of leaf gas exchange, nitrogen and nitrogen-use efficiency parameters for C_3_ × C_4_ hybrids and their parents grown in plant growth chambers Means ± SE. *n*=3–6 for gas exchange except for *A. rosea* (*n*=2). Abbreviations: ATPR, *A. prostrata*; ATRO, *A. rosea*; *A*_400_, net CO_2_ assimilation rate at an ambient CO_2_ of 400 µmol mol^–1^; *A*_max_, net CO_2_ assimilation rate at 800 µmol mol^–1^ CO_2_; C_i_/C_a_, ratio of intercellular to ambient CO_2_ concentration; N, nitrogen. Carboxylation efficiency is equal to the initial slope of the *A* versus *C*_*i*_ response. Superscripted x, y, or z indicate differences at *P*<0.05 between the ATPR, ATRO, F_1_ hybrid, and the pooled mean of all F_2_ hybrids. The a, b, c, or d letters after each value indicate statistical groups at *P*< 0.05 when all genotypes were compared. Statistical differences were tested using a one-way ANOVA followed by a Student-Newman-Keuls post-hoc test.

	*A*_400_	*A*_max_	C_i_/C_a_ @ 400	CO_2_ compensation point (Γ)	Carboxylation efficiency	Leaf N content	Leaf nitrogen-use efficiency (NUE) (=*A*_400_/leaf N)
Genotype	µmol m^–2^ s^–1^	µmol m^–2^ s^–1^	mol mol^–1^	µmol mol^–1^	mol m^–2^ s^–1^	mmol m^–2^	mmol mol^–1^ s^–1^
ATPR-C_3_	31.6±1.2a^x^	37.7±0.7a^x^	0.80±0.02a^x^	50.5±0.3a^z^	0.191±0.007b^y^	175±12a^x^	182±7ab^x^
ATRO–C_4_	31.2±0.0a^x^	32.8±0.6abc^xy^	0.57±0.09b^y^	–2.2±0.2d^x^	0.783±0.085a^x^	143±14a^x^	221±22a^x^
F_1_	16.0±1.0bc^y^	25.6±2.0c^y^	0.81±0.01a^x^	32.1±4.9c^y^	0.065±0.008c^z^	156 (n=1)	115 (N=1)
F_2_-107	20.2±1.1bc	31.2±1.1bc	0.80±0.02a	25.1±1.5c	0.100±0.007c	130±7a	131±5b
F_2_-108	17.9±1.0bc	30.7±1.0bc	0.76±0.02a	27.8±1.2c	0.088±0.004c	164±14a	110±19b
F_2_-109	20.5±0.8bc	32.1±0.9abc	0.81±0.01a	31.1±2.3c	0.094±0.004c	130±18a	164±26ab
F_2_-112	15.3±2.1c	27.7±2.8bc	0.79±0.07a	31.1±1.5c	0.069±0.007c	147±25a	104±47b
F_2_-114	16.0±0.4bc	26.6±0.3c	0.80±0.01a	3.8±2.4d	0.061±0.004c	125±4a	125±3.6b
F_2_-118	16.2±1.0bc	25.4±1.3c	0.76±0.06a	31.2±1.1c	0.084±0.007c	133±6a	124±15b
F_2_-119	15.9±3.1bc	28.6±4.2bc	0.71±0.06a	38.3±0.8bc	0.077±0.010c	141±15a	110±16b
F_2_-120	17.5±1.1bc	26.6±1.2c	0.82±0.02a	26.5±3.8c	0.083±0.004c	154±6a	115±8b
F_2_-123	21.3±0.6b	33.5±0.7ab	0.81±0.01a	44.6±1.0ab	0.102±0.004c	149±7a	147±8b
All F_2_	18.0±0.5^y^	29.2±0.6^y^	0.79±0.01^x^	29.5±2.0^y^	0.08±0.003^z^	145±4^x^	126±5^y^

## Leaf nitrogen content and nitrogen-use efficiency

Although the C_4_ parent and all hybrids lines exhibited lower leaf nitrogen content than the C_3_ parent, none of their means were significantly different (Table 1). Differences in leaf nitrogen-use efficiency (NUE) between the C_3_ and C_4_ species could not be statistically resolved, whereas each hybrid line except F_2_-109 has a significantly lower NUE than the C_4_ parent (Table 1). On average, the mean NUE of all the F_2_-hybrids was 31% less than the C_3_ mean and 43% less than the C_4_ value.

## Enzyme activity

**Fig. 4.**
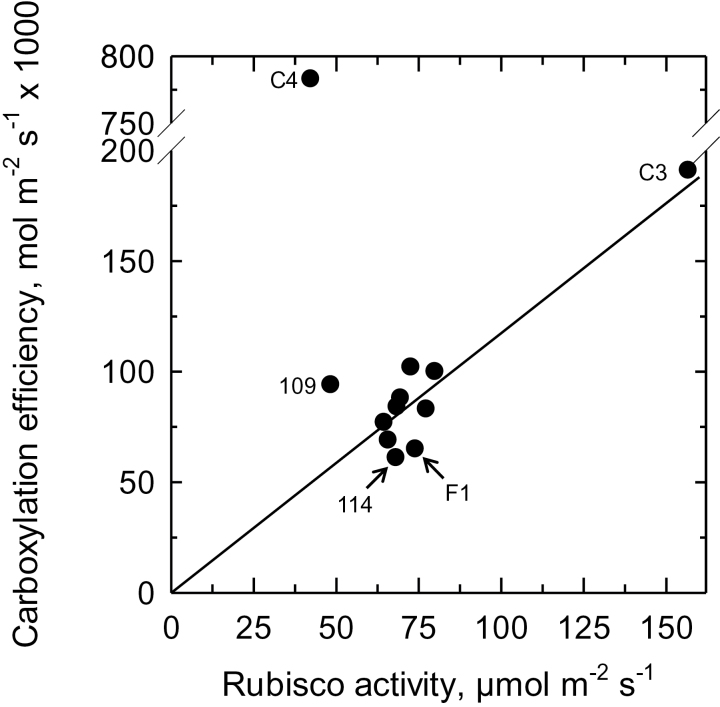
The carboxylation efficiency of photosynthesis as a function of *in vitro* Rubisco activity for the C_3_ species *Atriplex prostrata*, an F_1_ hybrid and all F_2_ hybrids in the study. Carboxylation efficiencies were calculated as the initial slope of the *A* versus *C*_i_ response for each genotype. Mean ± 3–6. The line is the theoretical carboxylation efficiency predicted for C_3_ Rubisco activity using the model of von Caemmerer (2000) and assuming the Rubisco activation state is 80%, Γ_*_ equals that of spinach at 30 °C, (Brooks and Farquhar, 1985) and the Rubisco kinetics and activation energies for the C_3_ *Atriplex glabriuscula* equal those of *A. prostrata* (von Caemmerer and Quick, 2000). “114” and “109” indicate the data points for F_2_-114 and F_2_-109.

The Rubisco activity of the F_1_ and F_2_ hybrids was 30–50% of the C_3_ value (Table 2). When the CE of each hybrid was plotted against its corresponding Rubisco activity, the hybrid values cluster around the theoretical relationship between Rubisco and CE in a C_3_ species (Fig. 4). The activities of the three major C_4_ cycle enzymes — PEPC, NAD-ME, and PPDK — were generally low in the hybrids and in many cases approached the activity of the C_3_ parent (Table 2). The F_1_ hybrid had significantly higher NAD-ME, PEPC, and PPDK activity than the C_3_ parent. Five of the nine F_2_ hybrids had significantly higher NAD-ME activities than the C_3_ parent, whereas just three had significantly higher PPDK activities than *A. prostrata.* Differences in PEPC between the F_2_ hybrids and the C_3_ parent could not be resolved using a one-way ANOVA at *P*<0.05; however, low statistical power in the test weakened our ability to resolve differences in PEPC activity. Four hybrids exhibited mean PEPC activities that were over twice the C_3_ value, and one of these, the F_2_-114 with the low, C_4_-like Г value, also had elevated activities of NAD-ME and PPDK (Table 2).

**Table 2.** The *in vitro* activity of NAD-malic enzyme (NAD-ME), PEP carboxylase (PEPC), pyruvate-phosphate dikinase (PPDK) and Rubisco at 30 °C Mean ± SE, *n*=4. Abbreviations: ATPR, *A. prostrata*; ATRO, *A. rosea.* Statistical differences between ATPR, ATRO, F_1_ and the pooled F_2_ means at *P*<0.05 were tested using one-way ANOVA followed by a Student-Newman-Keuls post-hoc test and are shown as superscripts x, y and z. * beside a value indicates means are significantly different from the ATPR activity using a one-way ANOVA followed by a Holm-Sidak post-hoc test where the ATPR mean was treated as the control value.

Genotype	Enzyme Activity, µmol m^–2^ s^–1^			
	NAD-ME	PEPC	PPDK	Rubisco
ATPR-C_3_	2.0±1.1^z^	12.5±1.4^z^	2.2±1.2^z^	156.7±5.1^x^
ATRO-C_4_	39.9±4.3^x*^	223.2±19.1^x*^	43.0±5.1^x*^	42.3±3.4^z*^
F_1_	7.8±0.8^y*^	55.9±7.9^y^*	16.0±0.6^y^*	74.0±6.3^y^*
F_2_-107	11.2±0.2*	32.9±6.3	3.1±1.4	79.9±6.8*
F_2_-108	11.8±1.1*	27.4±5.9	2.8±0.7	69.5±8.9*
F_2_-109	8.2±1.6	15.8±3.8	3.2±0.9	48.4±7.9*
F_2_-112	9.7±0.8*	15.3±4.7	4.0±1.7	65.7±12.5*
F_2_-114	9.6±1.3*	26.8±3.0	15.3±1.8*	68.2±5.4*
F_2_-118	4.5±1.8	20.7±2.1	11.7±3.7*	68.4±6.0*
F_2_-119	9.1±1.1	28.8±6.6	2.8±1.0	64.5±4.8*
F_2_-120	12.0±0.8*	27.7±2.0	3.7±0.6	77.3±2.1*
F_2_-123	4.6±0.7	23.9±5.4	13.2±3.3*	72.7±0.3*
All F_2_	9.1±0.6^y^	24.7±1.7^z^	6.9±1.1^z^	69.5±2.9^y^

## Leaf anatomy

**Fig. 5.**
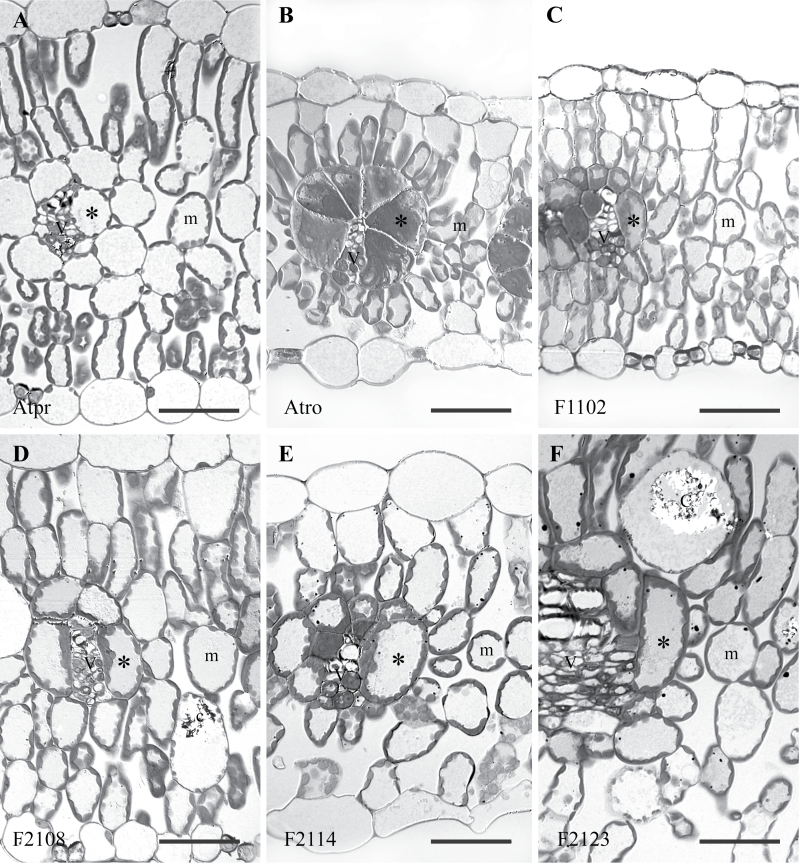
Light micrographs of cross-sections through leaves of (A) *Atriplex prostrata*, (B) *Atriplex rosea*, (C) their F_1_ hybrid, (D) F_2_-108, (E) F_2_-114, and (F) F_2_-123. See Supplementary Fig. S3 for light micrographs of leaf cross sections for the other six hybrids in the study. “*” delineates bundle sheath cells; C, a crystal containing cell; m, mesophyll cells; and V, vascular bundles. Bars=50 µm.

**Fig. 6.**
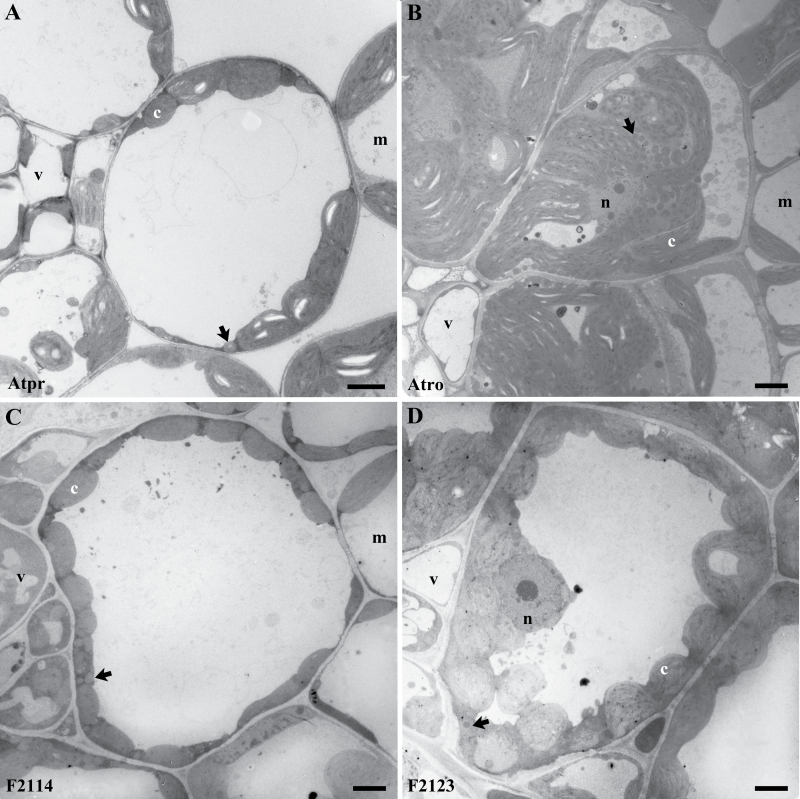
Transmission electron micrographs of bundle sheath cells in cross section of (A) *Atriplex prostrata*, (B) *Atriplex rosea*, (C) F_2_-114, and (F) F_2_-123. Arrows delineate mitochondria. Abbreviations: c, chloroplasts; m, mesophyll cells; n, nucleus; and v, vascular tissue. Bars=0.5 µm.

The leaf anatomy (Fig. 5A, B) and ultrastructure (Fig. 6A, B) of *A. prostrata* and *A. rosea* were typical for C_3_ and C_4_ members of the genus (Downton *et al.*, 1969; Dengler *et al.*, 1995). *Atriplex rosea* has well-developed BS cells that are discontinuous on the abaxial side of the vein (see also Liu and Dengler, 1994). In cross-section, BS cells are triangular in shape, which allows them to be tightly packed against the vein. Enlarged chloroplasts occupy the centripetal half of the BS cells in *A. rosea*, whereas no chloroplasts occur in the outer-most region of the cells (Figs. 5B, 6B). This is typical for the Atriplicoid-type of Kranz anatomy (Dengler and Nelson, 2000). In *A. prostrata*, BS chloroplasts are smaller than in the C_4_ plants and the chloroplasts are generally positioned along the outer periphery of the BS cell opposite intercellular air spaces. In cross section, chloroplasts were infrequent along the inner, centripetal wall of the BS cells of *A. prostrata*, and the individual BS cells were generally circular in outline. The BS cells of the F_1_ and F_2_ hybrids were variable in size and shape yet typically intermediate in structure between the C_3_ and C_4_ condition (Figs 5, 6, and Supplementary Fig. 3). Many of the BS cells of both F_1_ and F_2_ hybrids were oval in cross section, in contrast to the circular BS cells of *A. prostrata* and the triangular BS cells of *A. rosea*. This pattern resembles that observed in an immature leaf in *A. rosea* (see Fig. 4 in Liu and Dengler, 1994). In all hybrids, BS chloroplasts were numerous and arrayed all around the BS cell periphery (Figs 5 and 6). Chloroplast size and shape in the BS of the F_2_ hybrids was similar to what was observed in the BS of *A. prostrata* (Figs 5 and 6). In the BS cells of the hybrids, mitochondria occurred between chloroplasts, but did not form distinct ranks between elongated chloroplasts as observed in *A. rosea* (Fig. 6).

## Discussion

Results from this study and previous research with C_3_ × C_4_ hybrids demonstrate that C_4_ photosynthesis is disrupted in the hybrids, as shown by a general increase in the Γ, a reduction in CE and NUE, and the expression of a C_3_-like δ^13^C (Björkman *et al.*, 1971b, Björkman, 1976; Osmond *et al.*, 1980; Brown and Bouton, 1993). In the hybrids generated here, we observed that the F_1_ and most F_2_ hybrids exhibited Γ values in the mid-range between C_3_ and C_4_ species. However, one F_2_ line (#114) exhibited Γ values that overlap with those of C_4_-like species such as *Flaveria brownii* that have a fully functional C_4_ cycle (Ku *et al.*, 1991). A second F_2_ (#123) had Γ approaching the C_3_ range. In previous studies, F_1_ hybrids exhibit intermediate Γ values; these were interpreted to reflect a mix of C_3_ and C_4_ biochemistry in the F1 leaf (Pearcy and Björkman, 1971). The F_1_ hybrids are diploid with one set of chromosomes from each parent, and therefore have one C_3_ copy and one C_4_ copy of each gene, resulting in the mixed physiology (Osmond *et al.*, 1980). In F_2_ lines, trait segregation is apparent, and hybrids probably lose one or more of the genes essential for C_4_ function (Osmond *et al.*, 1980; Brown and Bouton, 1993). In F_3_ lines, further segregation leads to most hybrids exhibiting C_3_-like photosynthetic characteristics (Björkman *et al.*, 1971a, b). Occasionally, however, F_3_ hybrids exhibit Γ values close to the C_4_ value (Björkman, 1976), which is consistent with results from F_2_-114. Previous hybrid studies indicate that all parts of the C_4_ biochemical cycle and Kranz anatomy must be present for efficient C_4_ function (Björkman, 1976; Brown and Bouton, 1993). As these traits independently segregate (Brown and Bouton, 1993), the probability of an F_2_ hybrid acquiring all of the C_4_ traits is low, and hence, it is unlikely that full C_4_ photosynthesis can occur. However, Γ values in the mid-range between C_3_ and C_4_ plants demonstrate the existence of a CCM in the F_2_ lines. This could result from either a modest C_4_ metabolic pump or a C_2_-type CCM where photorespiratory glycine is shuttled into the BS cells (Brown and Bouton, 1993). With new hybrids, we are now in a position to evaluate these possibilities and develop working hypotheses to guide future hybrid studies. In our discussion of the F_2_ hybrids, we mainly focus on F_2_-114, whose C_4_-like Γ value indicates greater CCM activity.

In F_2_-114, the low, C_4_-like Γ is indicative of significant C_4_ cycle activity and/or a highly effective C_2_ CCM. F_2_-114 had activities of PEPC, PPDK and NAD-ME that were 12–30% of the C_4_ values, indicating the potential for a modest C_4_ cycle that could contribute to a reduction in Γ by supplying some CO_2_ to the BS. All other F_2_ hybrids in this study had C_3_-like activities in at least one of these enzymes, indicating low potential for more than minor C_4_ cycle activity. As shown by Type II C_3_-C_4_ intermediates (those with significant C_2_ photosynthesis and C_4_ metabolism; Edwards and Ku, 1987), modest C_4_ cycle activity combined with a C_2_-type of glycine shuttle is sufficient to reduce Γ below 10 µmol mol^–1^. In the Type II C_3_-C_4_ intermediate *F. ramosissima*, for example, a Γ of 7 µmol mol^–1^ was associated with C_4_ enzyme activities between 12% and 18% of C_4_ values (Ku *et al.*, 1983). A 3.5‰ increase in δ^13^C in F_2_-114 relative to *A. prostrata* is also evidence for modest C_4_ cycle activity, and is consistent with observed δ^13^C values of Type II intermediates such as *F. ramossissima*, and with modelled increases in δ^13^C assuming a 20–30% contribution by PEPC to the BS CO_2_ pool and moderate CO_2_ leakage (Monson *et al.*, 1988; von Caemmerer, 1992; Sudderth *et al.*, 2007). However, C_4_ metabolism could not contribute a large amount of carbon to the final pool of photosynthate in F_2_-114, because the δ^13^C values would shift more towards the C_4_ values than observed (von Caemmerer, 1992). We therefore hypothesize that the low Γ in F_2_-114 reflects a large contribution of a glycine shuttle to the CO_2_ pool of its BS cells.

Because C_2_ species with no C_4_-cycle activity (the type I C_3_-C_4_ intermediates; Edwards and Ku, 1987) exhibit Г values above 15 µmol mol^–1^ (Edwards and Ku, 1987; Ku *et al.*, 1991; Vogan *et al.*, 2007) it seems unlikely that a C_2_-type of glycine shuttle could reduce Γ to 4 µmol mol^–1^ by itself. However, according to von Caemmerer’s model of C_3_-C_4_ intermediate photosynthesis (von Caemmerer, 1989), a C_4_-like Γ could occur in a pure C_2_ species if there is an elevated (20%) fraction of leaf Rubisco in the BS cells, the conductance to CO_2_ leakage in the BS is low, and nearly all of the photorespired CO_2_ is released into the BS cells. Given the segregation of traits in the F_2_ lines (Osmond *et al.*, 1980), it is probable that these criteria could be met in a few hybrids. All of the F_2_ hybrids here exhibited Rubisco activities that are a third to a half that of the *A. prostrata* parent, indicating some C_4_-type control over Rubisco expression is present in the hybrid lines. C_4_ species produce 25–35% as much Rubisco as C_3_ species (Sage *et al.*, 1987), as is demonstrated by lower Rubisco activity in *A. rosea* relative to *A. prostrata*. Although we do not know where the Rubisco is distributed in our hybrids, previous work demonstrates F_1_ and F_3_ hybrids of *A. rosea* × *A. prostrata* express Rubisco in all chlorenchymatous cells (Hattersley *et al.*, 1977). The high number of plastids in the BS of the F_2_ hybrids also indicates significant amounts of Rubisco are present in their BS chloroplasts. With respect to BS conductance, we hypothesize that some hybrids, perhaps including F_2_-114, have inherited traits contributing to low, C_4_-like conductance in the BS, such as thick BS cell walls (von Caemmerer and Furbank, 2003). It is also likely that there is a high fraction of photorespiratory CO_2_ released in the BS of most hybrids. In C_4_ plants, photorespiratory glycine decarboxylase (GDC) is localized to BS cells, whereas in C_3_ plants, GDC and the photorespiratory cycle is expressed in both BS and M tissues (Muhaidat *et al.* 2011; Sage *et al.* 2011; Schulze *et al.* 2013). In an F_2_ hybrid, there is a good chance that one or more of the GDC subunits exhibit a C_4_ pattern and are not expressed in the M cells, whereas their expression in the BS cells would occur if either the C_4_ or C_3_ pattern were inherited. Hence, it is probable that GDC activity is low in the M tissues of the F_2_ hybrids and high in the BS, so that much of the photorespiratory glycine would have to migrate into the BS for decarboxylation. This would explain why most of the F_2_ lines have C_2_-like Γ values. Certain lines, such as F_2_-123 with a more C_3_-like Γ may have a leakier BS or relatively less Rubisco in the BS, whereas other lines with low Γ such as F_2_-114 may have proportionally more BS Rubisco or less BS leakiness, plus some contribution from a C_4_ cycle. These possibilities point to a need for enzyme localization and leakage assessments in future hybrid studies.

In most hybrids, it is apparent that the BS Rubisco is adequately supplied with CO_2_. When carboxylation efficiency is plotted as a function of Rubisco activity, the hybrid values clustered around the theoretical relationship between Rubisco activity and carboxylation efficiency of a C_3_ *Atriplex*-like plant (Fig. 4). This demonstrates that in most hybrids, Rubisco is on average operating with the same efficiency as in a C_3_ leaf. The CE of F_2_-109 sits well above the CE versus Rubisco activity plot, which would occur if much of its Rubisco is in a CO_2_-enriched environment. The low PEPC and PPDK activity in F_2_-109 indicates the reduction of Γ below C_3_ values is predominately due to CO_2_ influx into the BS via C_2_ photosynthesis. Hybrid F_2_-114 exhibits the lowest CE relative to the theoretical CE versus Rubisco plot, demonstrating that at least some of its Rubisco is operating with reduced efficiency. Low CO_2_ levels in the BS would reduce CE, but this would not result in the low Γ value of F_2_-114 because Rubisco oxygenase activity would increase at low CO_2_ and raise Γ. Alternatively, Rubisco may be limited by low RuBP regeneration capacity, or a low activation state owing to a lack of Rubisco activase. Low RuBP regeneration might result if a C_4_ pattern of thylakoid protein expression corresponded to a C_3_ pattern of Calvin cycle expression, in which case one of the C_3_ compartments could be energy limited. The potential lack of activase expression is an intriguing possibility that could not be considered in the first era of *Atriplex* hybrid studies, as activase was unknown at the time. In C_4_ plants, activase expression is four times higher in the BS than M tissue (Majeran *et al.*, 2005). In the hybrids, a C_4_-like pattern of activase expression could leave Rubisco in the M cells in a partially deactivated state. This would explain the low CE in F_2_-114, as the M Rubisco could be deactivated and unable to contribute to the CE values.

### Anatomical patterns

Anatomically, all of the hybrid lines failed to express the well-developed Atriplicoid-type of Kranz anatomy, as has been noted before (Boynton *et al.*, 1970). Atriplicoid Kranz anatomy consists of enlarged BS cells with a surrounding layer of M cells (Liu and Dengler, 1994; Dengler and Nelson, 1999). Chloroplasts in the BS cells of *A. rosea* are elongated and fill the inner two-thirds of the BS, and have many mitochondria distributed along the sides of the chloroplasts (Fig. 6). No chloroplasts or mitochondria occur along the outer BS wall of C_4_ *Atriplex* species. This arrangement allows for rapid re-assimilation of CO_2_ released by NAD-ME in the mitochondria, with the vacuole of the outer BS providing significant resistance to CO_2_ efflux (von Caemmerer and Furbank, 2003). By contrast, the C_3_ *A. prostrata* produces small BS chloroplasts that are similar to M cell chloroplasts; these occur along the outer wall of the BS cell against the intercellular air spaces (Boynton *et al.*, 1970). In all the hybrids, the BS chloroplasts are similar in size and shape to those of the C_3_ parent, yet their positioning resembles a pattern that is often observed in C_2_-type species, where chloroplasts can occur in both a centripetal and centrifugal position (Muhaidat *et al.*, 2011; Sage *et al.*, 2013). Mitochondria still occur between chloroplasts, but to less of a degree than seen in *A. rosea*. Many of the mitochondria in the hybrids also appear between the inner BS wall and the chloroplasts, resembling a pattern apparent in C_3_-C_4_ intermediate plants using the C_2_-type of CCM (Monson and Rawsthorne, 2000; Sage *et al.*, 2011; 2013). These observations further indicate that the BS cells of the F_2_ hybrids use the C_2_ mode of photosynthesis, although this will depend upon whether enough GDC is present in the BS mitochondria to create a strong sink for glycine produced in the M tissue.

### Stomatal control

Previous work with C_3_ × C_4_ hybrids did not emphasize stomatal control, due in part to incomplete understanding at the time of stomatal regulation in C_3_ and C_4_ species. It is now known that non-stressed C_3_ species regulate *C*_*i*_/*C*_*a*_ to generally be between 0.7–0.8 under humid conditions, whereas in C_4_ plants, *C*_*i*_/*C*_*a*_ is maintained between 0.4–0.6 (Wong *et al.*, 1979; Taylor *et al.* 2011; Vogan and Sage, 2011). The lower *C*_*i*_/*C*_*a*_ in C_4_ species reflects tighter stomatal control and increased carboxylation efficiency of the C_4_ pathway relative to C_3_ photosynthesis; this explains the greater water-use efficiency of C_4_ plants (Huxman and Monson, 2003; Vogan and Sage, 2011). Under the relatively low vapour pressure difference between leaf and air in this study, we observed *C*_*i*_/*C*_*a*_ to be 0.57 in *A. rosea* and 0.80 in *A. prostrata.* In the hybrids, the *C*_*i*_/*C*_*a*_ values have largely reverted to the C_3_ value (*C*_*i*_/*C*_*a*_ of 0.71–0.81), indicating that a full complement of C_4_ machinery is required for a C_4_ pattern of stomatal control.

## Conclusions

With the new C_3_ × C_4_ hybrids in *Atriplex*, we have re-established an important system for investigating the genetic control and physiological function of C_4_ photosynthesis. In the F_2_ lines, we demonstrate a loss of efficient C_4_ function, further supporting the hypothesis that all of the components of the C_4_ pathway must be in place for C_4_ photosynthesis to occur. Although impairment of C_4_ photosynthesis in the F_2_ hybrids is no surprise, an intriguing observation is that improper assembly of the C_3_ pathway is also apparent in most F_2_ hybrids. This may reflect incomplete expression of the photorespiratory pathway in the M cells of the hybrids, or mismatched compartmentalization of C_3_ photosynthetic components. Ironically, with the incomplete assembly of the C_3_ and C_4_ conditions in the F_2_ lines, the default state seems to be C_2_ photosynthesis, for what may be a rather simple reason. Because both C_3_ and C_4_ plants express GDC in the BS (Schulze *et al.*, 2013), the probability is high that GDC of the F_2_ hybrids is abundant in the BS cells, whereas GDC levels in the M cells may be low owing to inheritance of C_4_ expression patterns for at least one of the four GDC subunits. Hence, glycine would have to flow to the BS for decarboxylation, to the benefit of Rubisco in the BS chloroplasts.

We have now successfully generated the F_3_ hybrids and will be producing F_4_ lines and beyond to further segregate traits and possibly create near isogenic lines. With the analytical capabilities provided by modern tools and theory, we are better positioned to evaluate genetic, biochemical, and structural limitations affecting photosynthesis in the hybrids and hence provide critical information that can be utilized to engineer C_4_ photosynthesis into C_3_ crops as well as understand the evolution of C_4_ photosynthesis. These were the initial goals of Olle Björkman, John Boynton, Malcolm Nobs, and Bob Pearcy in the late 1960s when the initial hybrids were created. In the near future, these goals may be realized.

## Supplementary data

Supplementary data are available at *JXB* online.

Supplementary Figure S1. Photographs of the *Atriplex* parents, F1 hybrid and F2 hybrids from this study.

Supplementary Figure S2. The response of net CO_2_ assimilation rate to intercellular CO_2_ partial pressure for the *Atriplex* parents and C_3_ x C_4_ hybrids in this study.

Supplementary Figure S3. Light micrographs of cross- sections through leaves of six *Atriplex prostrata* x *Atriplex rosea* F_2_ hybrids from this study.

Supplementary Data
