# Supplementary material for: Comparative studies of C3 and C4 Atriplex hybrids in the genomics era: physiological assessments
Source: J Exp Bot. 2014 Mar 27;65(13):3637–47. doi: 10.1093/jxb/eru106 (PMC4085961; doi:10.1093/jxb/eru106)
Supplement: Supplementary Data [file supp_65_13_3637__index.html]

Comparative studies of C3 and C4 Atriplex hybrids in the genomics era: physiological assessments — Comparative studies of C3 and C4 Atriplex hybrids in the genomics era: physiological assessments — Supplementary Data 

# Comparative studies of C3 and C4*Atriplex* hybrids in the genomics era: physiological assessments

## Supplementary Data

Data files

**Files in this Data Supplement:**

- Supplementary Data - Supplementary Data
