# Supplementary material for: Comparative studies of C3 and C4 Atriplex hybrids in the genomics era: physiological assessments
Source: J Exp Bot. 2014 Mar 27;65(13):3637–47. doi: 10.1093/jxb/eru106 (PMC4085961; doi:10.1093/jxb/eru106)
Supplement: Supplementary Data [file supp_eru106_jexbot117325_file001.pdf]

Supplementary Figure S1. Photographs of the *Atriplex* parents, F_1 hybrid, and F_2 hybrids included in this study. Composite of four-week old plants (A) and leaves (B) taken from the same plants. Leaf photographs are of the second most-recently fully expanded leaf from the youngest axillary shoot.

Supplementary Figure S2. The response of net CO_2 assimilation rate, *A*, to intercellular CO_2, C_i. All *A versus* C_i response curves were measured at 30°C and light intensity of 1500 µmol photons m^{-2} s^{-1}. (A) The parents and F_1 hybrid. (B) F_2 hybrids with CO_2 compensation point (Γ) < 30 µmol mol^{-1}. (C) F_2 hybrids with Γ > 30 µmol mol^{-1}.

Supplementary Figure S3. Light micrographs of cross-sections through leaves of *Atriplex prostrata* x *Atriplex rosea* hybrids (A) F_2-107, (B) F_2-109, (C) F_2-112, (D) F_2-118, (E) F_2-119, and (F) F_2-120. Abbreviations: *, bundle sheath cells; c, chloroplasts, m, mesophyll cells, n, nucleus, and v, vascular tissue. Bars = 50 µm.

Oakley et al, Supplemental Fig. S1

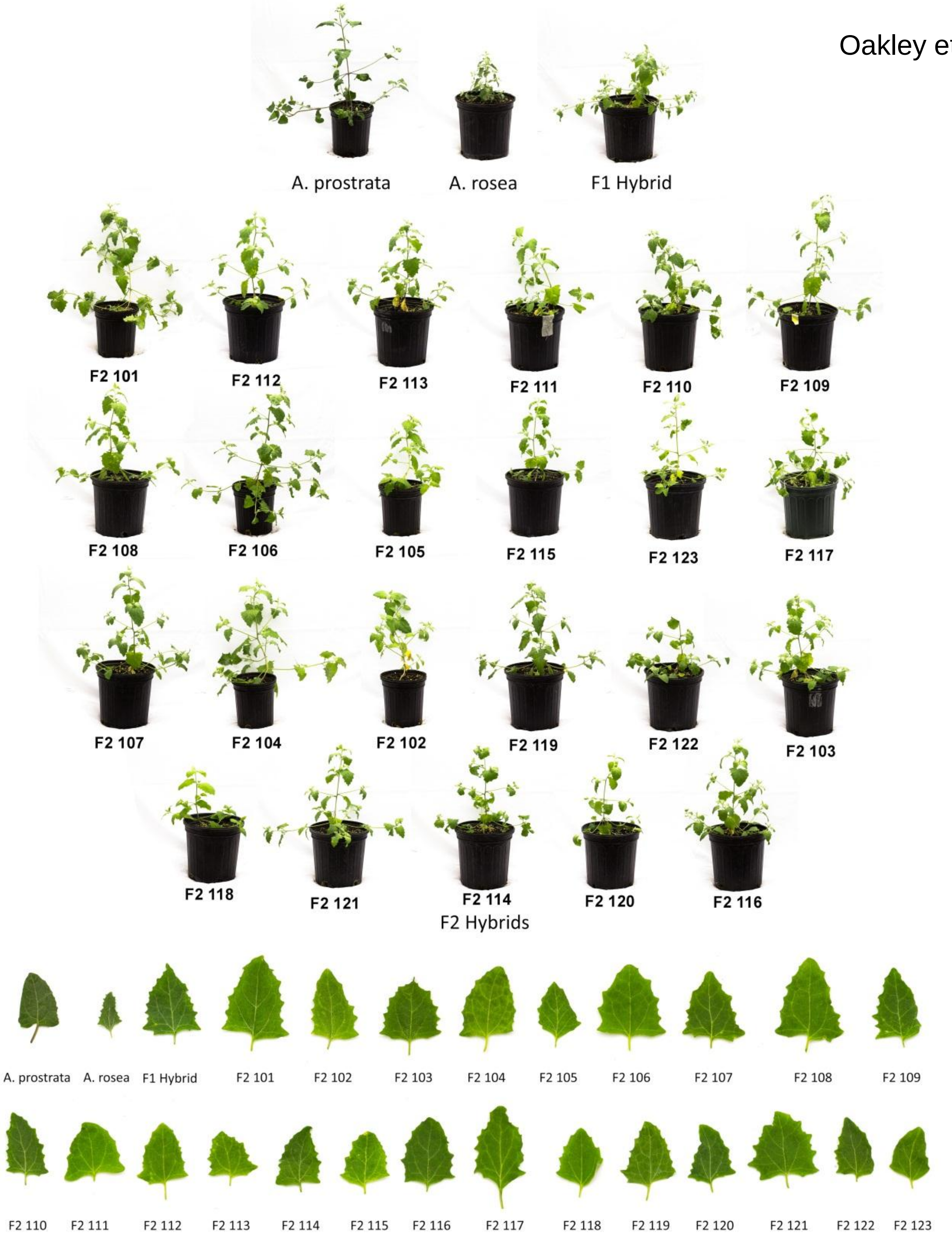

Oakley et al, Supplemental Fig S2

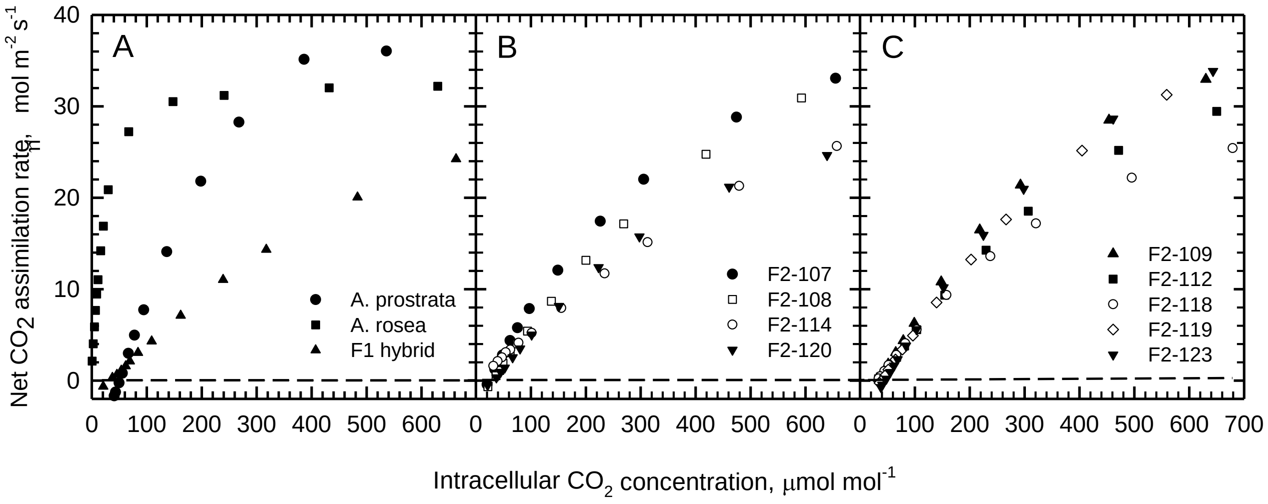

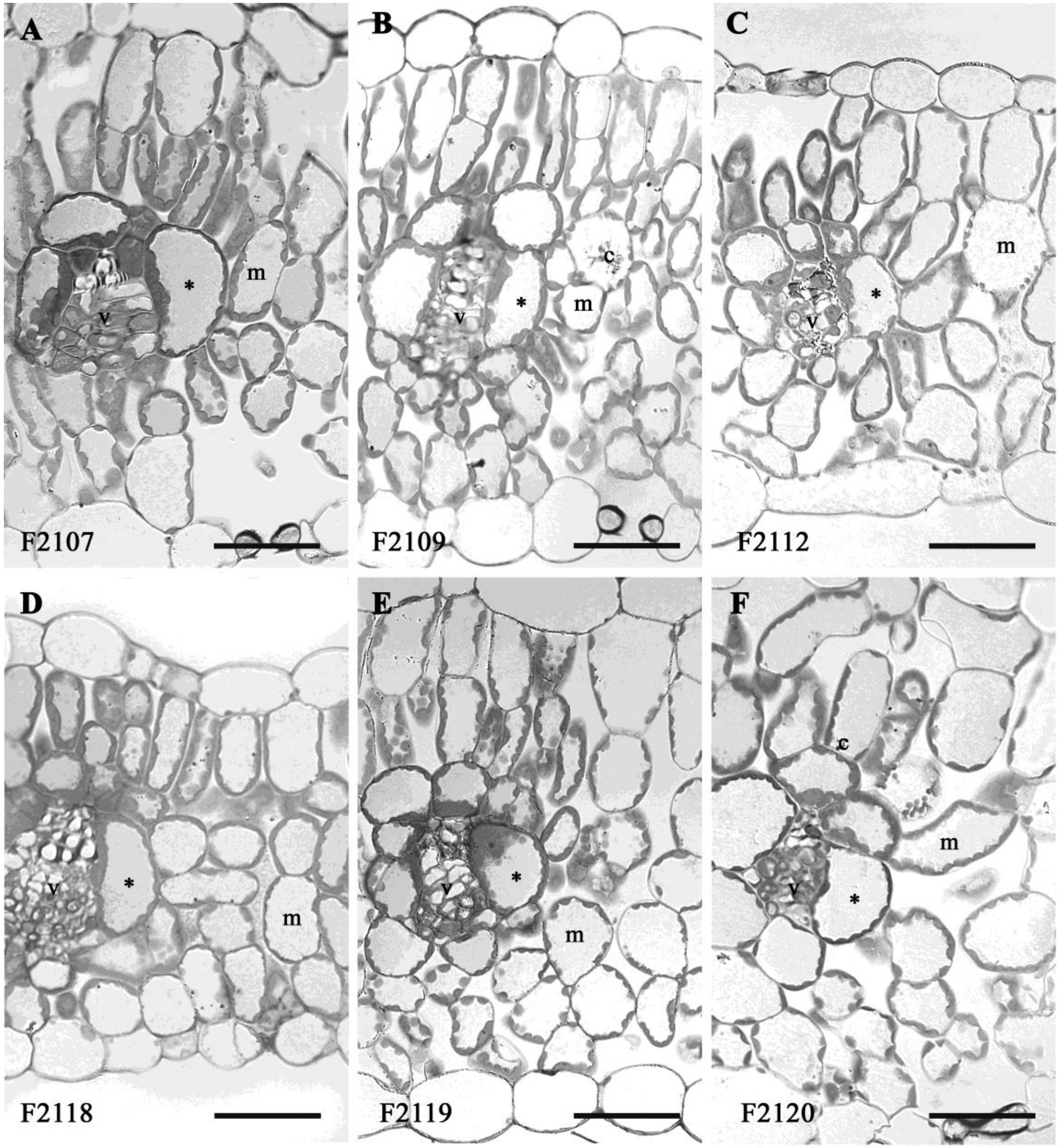
A
m
*
v
F2107
B
c
*
m
v
F2109
C
m
*
v
F2112
D
v
*
m
F2118
E
v
*
m
F2119
F
c
m
v
*
F2120
